# Bioinformatics analysis and experimental validation of TTK as a biomarker for prognosis in non-small cell lung cancer

**DOI:** 10.1042/BSR20202711

**Published:** 2020-10-06

**Authors:** Jiajia Chen, Rong Wu, Ying Xuan, Min Jiang, Yuecan Zeng

**Affiliations:** Department of Clinical Oncology, Shengjing Hospital of China Medical University, Shenyang, China

**Keywords:** biomarkers, differentially expressed genes, overall survival, single-cell RNA sequencing

## Abstract

**Background:** Despite the prominent development of medical technology in recent years, the prognosis of non-small cell lung cancer (NSCLC) is still not optimistic. It is crucial to identify more reliable diagnostic biomarkers for the early diagnosis and personalized therapy of NSCLC and clarify the molecular mechanisms underlying NSCLC progression.

**Methods:** In the present study, bioinformatics analysis was performed on three datasets obtained from the Gene Expression Omnibus to identify the NSCLC-associated differentially expressed genes (DEGs). Immunohistochemistry-based tissue microarray of human NSCLC was used to experimental validating the potential targets obtained from bioinformatics analysis.

**Results:** By using protein–protein interaction (PPI) network analysis, Kaplan–Meier plotter, and Gene Expression Profiling Interactive Analysis, we selected 40 core DEGs for further study. Then, a re-analysis of 40 selected genes via Kyoto Encyclopedia of Genes and Genomes pathway enrichment showed that nine key genes involved in the cell cycle and p53 signaling pathway participated in the development of NSCLC. Then, we checked the protein level of nine key genes by semi-quantitative of IHC and checked the distribution at a single-cell level. Finally, we validated dual-specificity protein kinase TTK as a biomarker for prognosis in a tissue microarray. High TTK expression associated with a higher histological stage, advanced TNM stage, high frequency of positive lymph nodes, and worse 5-year overall survival.

**Conclusions:** We found nine key genes were enriched in the cell cycle and p53 signaling pathway. TTK could be considered as a potential therapeutic target and for the prognosis biomarker of NSCLC. These findings will provide new insights for the development of individualized therapeutic targets for NSCLC.

## Introduction

Lung cancer is one of the most prevalent cancers and the leading cause of cancer-related deaths in the world. More than one million deaths were caused by lung cancer worldwide annually. Lung cancer is classified into two major subtypes: small-cell lung cancer and non-small-cell lung cancer (NSCLC). NSCLC, includes adenocarcinoma (LUAC) and squamous cell carcinoma (LUSC), is the major pathological type of lung cancer, accounts for 80–85% of lung cancer cases [1]. Despite the prominent development of medical technology in recent years, the prognosis of lung cancer is still not optimistic with a poor overall 5-year survival rate of 10–15% [2]. This might be partly due to the difficulties for early diagnosis and non-effective therapeutic targets for patients with NSCLC. Since the lack of obvious signs and symptoms in the early-stage of NSCLC patients, the diagnosis of NSCLC is often made at an advanced stage [2,3]. Consequently, NSCLC patients bear poor prognosis, as the 5-year survival rate is 57% for patients with stage I disease and decreased to 4% for those with stage IV disease [1]. It is crucial to identify more reliable diagnostic biomarkers for the early diagnosis and personalized therapy of NSCLC and clarify the molecular mechanisms underlying NSCLC progression.

Gene expression in NSCLC is widely studied by RNA microarray that can quantify expression of all genes and is proved to be a powerful and reliable techniques [4]. A lot of expression profiles have been done in the NSCLC by using RNA microarray; however, they are not effectively explored. In recent years, bioinformatics has been widely utilized in oncology research to find the differential expressed genes (DEGs) [5]. DEGs may play a pivotal role in cancer development and are expected to be used for exploring significant diagnoses and more effective therapeutic strategies for NSCLC. Moreover, the interactions among the identified DEGs, the important signal pathways, and the protein interaction networks should be elucidated to understanding the molecular mechanism of NSCLC. Thus, it is important to explore the existing database and find and validate more effective targets for early diagnosis marker and therapeutic strategies for NSCLC.

In the present study, three original gene expression profile datasets (GSE31552, GSE43458, and GSE44077) were obtained from the Gene Expression Omnibus to identify the NSCLC-associated DEGs in the tumor tissue versus non-tumor lung samples [6–8]. A total of 284 DEGs were identified in the present study. In addition, we performed gene ontology (GO), Kyoto Encyclopedia of Genes and Genomes (KEGG) enrichment pathway analysis, and protein–protein interaction (PPI) network analysis to illustrate significant associations and functional information of identified DEGs. Furthermore, 40 DEGs have been confirmed to relevant to the prognosis of NSCLC by STRING analysis. By deeply KEGGs analysis, nine genes were selected as the potential targets for future treatment. This nine DEGs expression were also confirmed in RNA and protein levels, as well as in single-cell RNA sequencing level. Finally, we validated dual-specificity protein kinase TTK as a biomarker for prognosis in a tissue microarray.

## Materials and methods

### GEO data retrieval

The microarray data were retrieved from the GEO database (http://www.ncbi.nlm.nih.gov/geo) of the National Center for Biotechnology Information. In the present study, three gene expression profiles were utilized from the GEO database (GSE31552, GSE43458, and GSE44077). The detailed information about the three profiles is shown in Supplementary Table S1. All the three microarray data were run on the GPL6244 platform [Affymetrix Human Gene 1.0 ST Array, transcript (gene) version]. All the background and the official gene symbols were the same. Totally, we have 105 non-tumor lung tissues and 155 NSCLC tissues.

### DEGs screening

DEGs between NSCLC tissues and normal tissues were identified by using GEO2R (http://www.ncbi.nlm.nih.gov/geo/geo2r/) for each individual datasets. The cut-off criteria were set as the adjusted *P* value<0.05 and |log FC| >1. Then, the raw data from each profile were checked in Venn software online v2.1 to get the commonly DEGs among the three profiles (https://bioinfogp.cnb.csic.es/tools/venny/). The DEGs with log FC<0 were supposed as down-regulated genes, while the DEGs with log FC>0 were regarded as up-regulated genes.

### GO and KEGG pathway enrichment analysis

In order to classify the function and interaction of identified DEGs, the Database for Annotation, Visualization, and Integrated Discovery (DAVID; version 6.8; david.ncifcrf.gov/) was used to performing GO enrichment and KEGG pathway analysis [9,10]. For GO enrichment, biological process (BP), cellular component (CC), and molecular function (MF) were analyzed. The adjusted *P* value (FDR) was analyzed by the Benjamini test. FDR<0.05 was considered to be statistically significant for BP, CC, MF, and KEGG processes.

### PPI network analysis

For interaction between DEGs, PPI information was retrieved by Search Tool for the Retrieval of Interacting Genes (STRING, string-db.org/) as previously described [11]. Briefly, a tool for multiple human proteins by names was applied to analyze all DEGs. The most significantly linked DEGs were selected for further analysis.

### Survival analysis by Kaplan–Meier plotter for NSCLC

The Kaplan–Meier plotter is explored to analyze the effect of 54k genes on survival in 21 cancer types. The database involves gene chip and RNA-sequencing data sources and the databases include data from GEO, European Genome-Phenome Archive (EGA), and Cancer Genome Atlas (TCGA). The primary purpose of the tool is a meta-analysis based discovery and validation of survival biomarkers [12]. The Kaplan–Meier plotter was used to evaluate the effect of selected DEGs on the survival of NSCLC. The log-rank *P* value and hazard ratio (HR) with 95% confidence intervals were computed and showed on each plot. Log-rank *P*<0.05 was considered to be statistical significant.

### Validation of the mRNA expression of identified DEGs

The Gene Expression Profiling Interactive Analysis (GEPIA) is a recently established web-based tool for gene expression analysis between normal tissues and tumor tissues [13]. Based on the TCGA and Genotype-Tissue Expression (GTEx) database, GEPIA collected the expression data of 486 cases of LUSC and 483 cases of LUAD as well as corresponding normal tissues. We checked the identified DEGs expression both in LUSC and LUAD. *P*<0.05 was considered to be statistical significant. Additionally, the mRNA levels of identified DEGs were also revealed in three lung cancer cell lines (A549, HBEC3-KT, and SCLC-21H) by using Human Protein Atlas (HPA, www.proteinatlas.org).

### Validation of the protein level of identified DEGs

Human protein Atlas was used to show the immunohistochemistry (IHC) staining of selected DEGs [14,15]. The staining and intensity of each protein were also shown in the database. The expression of the protein in the tissues was scored semi-quantitatively by combining the staining and the intensity (staining index = staining × intensity), according to previous studies [16]. Briefly, the staining of the IHC image was scored as 0, not detected; 1, low; 2, moderate; 3, high. The intensity of the IHC image was graded on the following scale: 0, negative; 1, weak; 2, moderate; 3, strong staining. Then, the staining index of normal lung tissues and NSCLC tissues was quantified and analyzed by using *t*-test. *P*<0.05 was considered to be statistical significant.

### Distribution of identified genes in single-cell level

For the heterogeneity of gene expression in NSCLC, we confirmed the selected genes in single-cell RNA-sequencing based database [17]. Seven lung tumor samples were dissociated for single-cell sequencing and re-clustered into a natural killer (NK) cells T cells, B cells, monocyte/macrophage/dendritic cells (DC), neutrophils, plasma cells, and other cells by using specific cell markers. We checked the distribution of nine key genes in total human tumor cells from the website tool (https://kleintools.hms.harvard.edu/tools/springViewer_1_6_dev.html?datasets/Zilionis2019/human/NSCLC_all_cells). We also confirmed these data in another single-cell RNA-sequencing based database which include 3 lung tumor samples (https://www.ebi.ac.uk/gxa/sc/experiments/E-MTAB-6653/).

### Tissue microarray of human NSCLC

Human NSCLC tissue microarray was obtained from National Engineering Center for Biochip at Shanghai (Shanghai, China). The TMA contains 90 cases of the NSCLC tissue that were collected from patients who underwent complete surgical resection of tumors between May 2008 and September 2013. The clinical parameters were collected, and patients were follow up after surgery to June 2016. All the tissue samples were fixed with formalin, embedded in paraffin, and sectioned by the manufacturer. Written informed consent forms were given and signed by all patients before participation. The study was approved by the ethical committee of Shengjing Hospital, China Medical University.

### Immunohistochemistry

Tissue microarray sections were deparaffinized in xylene and rehydrated in a series of ethanol dilutions. Antigen retrieval was performed by heating sections with 10 mM sodium citrate buffer. Subsequently, sections were blocked with 10% goat serum and then incubated with primary TTK antibody (1:100, Proteintech #10381-AP) overnight at 4°C. Next, sections were incubated with secondary antibody for 30 min, stained with 3, 3′-diaminobenzidine tetrahydrochloride solution, and counter-stained with hematoxylin.

### IHC scoring

Two independent pathologists blinded to clinical and pathologic information assessed the expression of TTK on the tissue microarray slide. The expression of TTK in NSCLC tissues was scored semi-quantitatively combining positive percentage and intensity of stained sections (staining index = positive × intensity score) according to our previously described. Based on the staining index, a final total score of top 50% cases were considered to be high TTK group, whereas others were defined as low TTK group.

### Statistical analysis

Quantitative data were expressed as mean ± standard deviation. The Kaplan–Meier method was utilized to depict survival curves that were compared by the log-rank test. The χ^2^ test was applied to analyze categorical variables. The receiver operating characteristic (ROC) curve was plotted by SPSS 19.0 with the area under the ROC curve (AUC) ranges from 0.5 and 1.0 indicative of better fit. All data were analyzed by SPSS 19.0 software (SPSS, Chicago, IL, U.S.A.). A *P*-value of < 0.05 was considered statistically significant.

## Results

### Identification of DEGs in NSCLC

**Figure 1:**
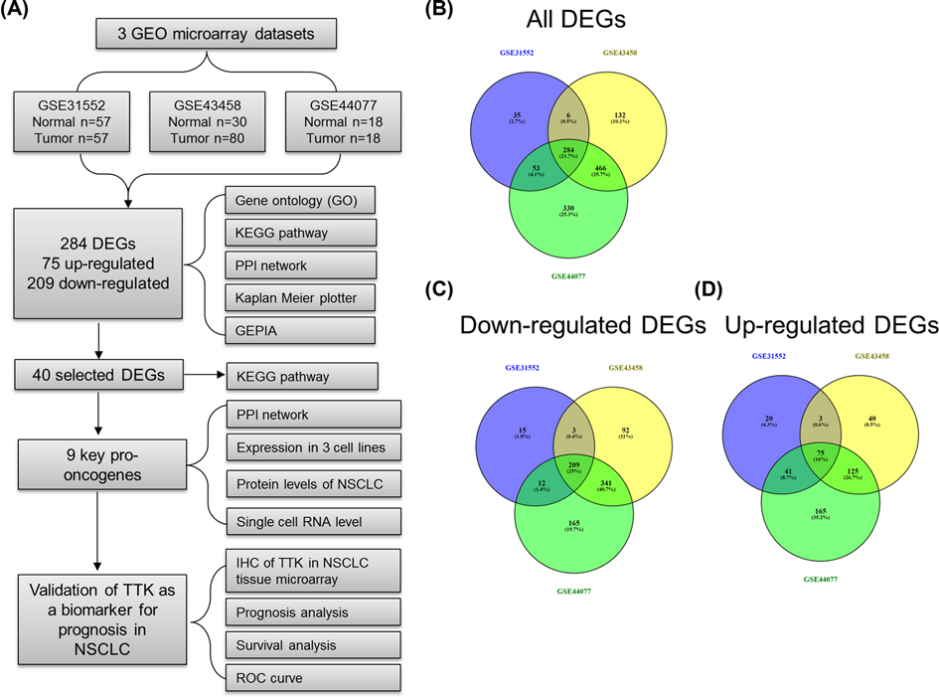
Overlap of mRNA expression profiling in GSE31552, GSE43458 and GSE44077 The flowchart of bioinformatics analysis (**A**). The Venn diagram reveals all DEGs (**B**), down-regulated DEGs (**C**) and up-regulated DEGs (**D**). The overlapped areas show the number of DEGs among GSE31552, GSE43458, and GSE44077.

The flowchart of the present study was shown in Figure 1A. All three microarrays were performed on the same platform GPL6244 (Supplementary Table S1). Totally, there were 155 NSCLC tissues and 105 normal tissues included in the present study. In GSE31552, 57 adenocarcinomas/squamous cell carcinoma and 57 corresponding non-tumor tissues were included. In GSE43458, 80 adenocarcinomas and 30 non-tumor tissues were analyzed. In GSE44077, 18 adenocarcinomas/squamous cell carcinoma and 18 corresponding non-tumor tissues were included. By using Venn online software, 378, 888, and 1133 DEGs were extracted from GSE31552, GSE43458, and GSE44077 datasets, respectively. Furthermore, 284 DEGs were overlapped among three databases including 209 down-regulated DEGs (logFC>0) and 75 down-regulated DEGs (logFC<0) between NSCLC tissues and non-tumor tissues (Figure 1B–D). The official gene symbols were listed in Supplementary Table S2. In summary, we identified 284 DEGs in NSCLC.

### GO and KEGG pathway analysis of identified DEGs in NSCLC

DAVID software was used to analyze GO and KEGG pathway enrichment based upon 75 up-regulated DEGs and 209 down-regulated DEGs. For the GO analysis of 75 up-regulated DEGs, 11 BP, 13 CC, and 5 MF were enriched (Supplementary Table S3). The top processes were mitotic nuclear division, nucleoplasm, and ATP binding. For the GO analysis of 209 down-regulated DEGs, 3 BP, 6 CC, and 1 MF were enriched (Supplementary Table S4). The most enriched processes were angiogenesis, membrane rafts, and heparin-binding. In order to find the key signaling pathway, KEGG analysis was performed on 75 up-regulated DEGs and 209 down-regulated DEGs. For up-regulated DEGs, the cell cycle signaling pathway was enriched by KEGG analysis (FDR = 1.70E-5, *P*=3.30E-07, Supplementary Table S5). However, none of the signaling pathway was significantly enriched in down-regulated DEGs (FDR>0.05, Supplementary Table S5). In summary, the identified DEGs were important for multiple cellular processes.

### PPI network analysis of identified DEGs in NSCLC

**Figure 2:**
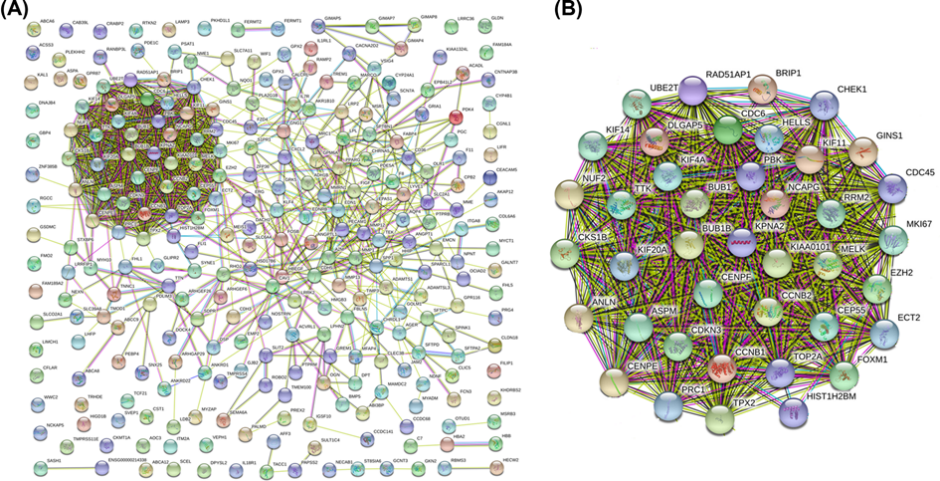
PPI networks of the identified DEGs (**A**) The PPI networks performed on STRING online database for all 284 DEGs in NSCLC. (**B**) The most significant PPI networks, 44 genes are enriched in this region.

STRING database was used to construct the PPI networks for investigation of the interactions between DEGs. There was one cluster that has a strong PPI network among proteins (Figure 2A). Then, the proteins in this region were selected for further analysis by which indicates this region contains 44 DEGs, including 40 up-regulated DEGs, and 4 down-regulated DEGs (Figure 2B). In summary, 44 DEGs were selected as core molecules that may contribute to NSCLC progress.

### Analysis of core DEGs by Kaplan–Meier plotter and GEPIA

**Figure 3:**
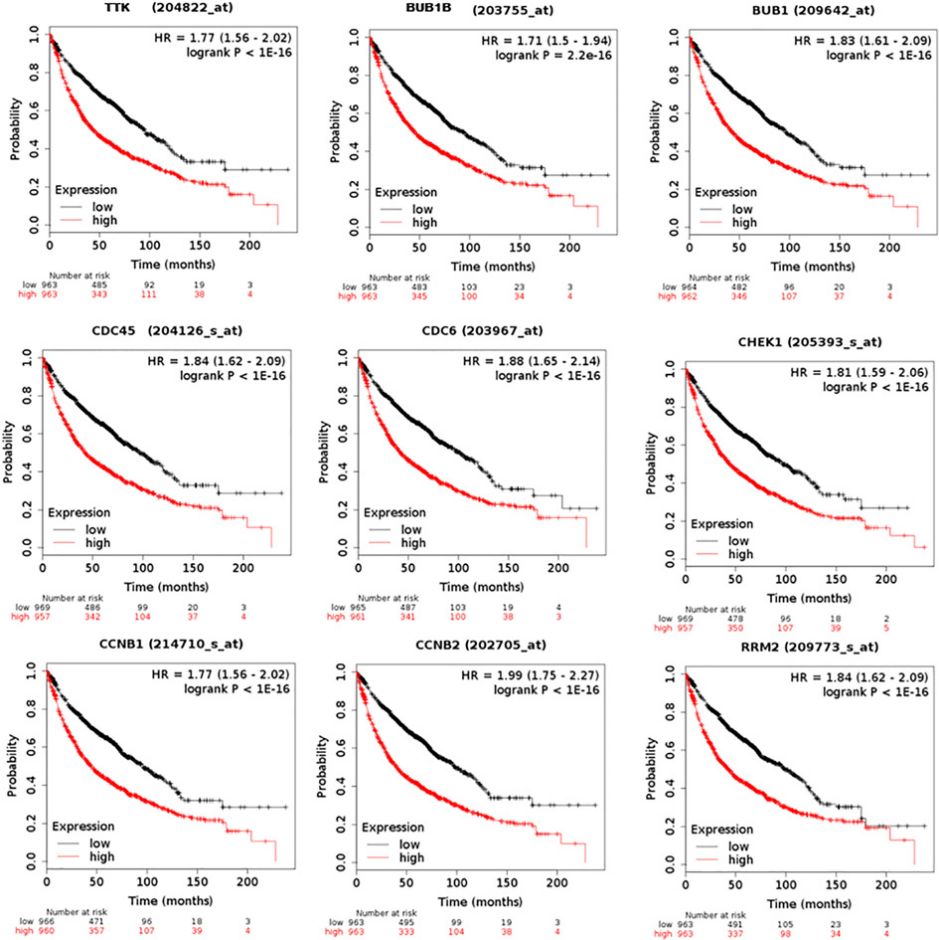
Overall survival of 44 core DEGs in NSCLC Kaplan–Meier plotter is applied to generating the survival curve for all nine core DEGs. Log-rank *P* value and hazard ratio (HR) with 95% confidence intervals are computed and showed on the plot. Log-rank *P*<0.05 is considered to be statistical significant.

**Figure 4:**
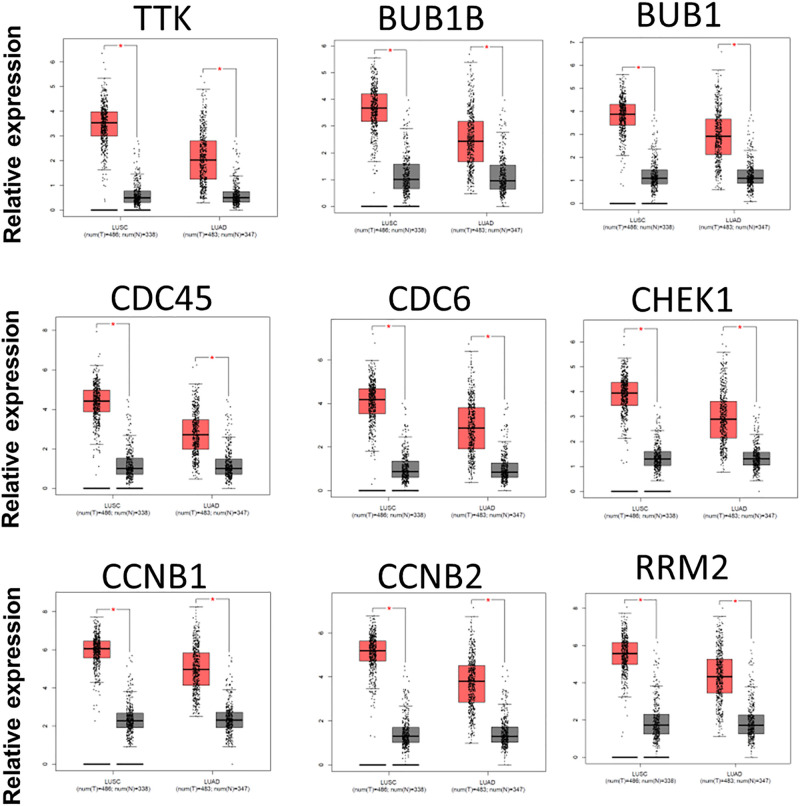
The mRNA levels of nine core DEGs in LUAD, LUSC, and the corresponding normal lung tissues Nine DEGs are up-regulated DEGs in LUAD and LUSC compared with corresponding normal lung tissues. The red color histogram on left reveals LUSC, the red color histogram on right shows LUAD, and the gray color histogram means the corresponding normal lung tissues; *P<0.05.

The 44 core DEGs were further evaluated by using the Kaplan–Meier plotter to indicating their prognostic values in NSCLC. The expression of 42 core DEGs were significantly correlated with overall survival of NSCLC patient (Figure 3; Supplementary Figure S1 and Supplementary Table S6). Of which, high expression of 39 core DEGs predicted poor prognosis in NSCLC, and low expression of 3 core DEGs revealed poor prognosis of NSCLC (Figure 3; Supplementary Figure S1 and Supplementary Table S6). Next, we confirmed the expression of core DEGs by using the GEPIA database. Except that the mRNA level of HIST1H2BM showed no significant difference between NSCLC and normal tissues, the mRNA levels of all other 43 core DEGs were significantly changed between NSCLC and normal tissues (Figure 4 and Supplementary Figure S2). The mRNA levels of 39 core DEGs were significantly increased in NSCLC tissues compared with that in normal lung tissues (Figure 4 and Supplementary Figure S2). Meanwhile, the mRNA levels of FOSB, SYNE1 TTN, and MYH10 were significantly decreased in both LUSC and LUAD compared with that in normal lung tissues (Supplementary Figure S2). Next, we analyzed the relationship between the expression of core DEGs and prognosis. We found that 38 up-regulated DEGs predicted poor prognosis, and 2 down-regulated DEGs correlated with good prognosis (Supplementary Table S7). However, SYNE1 showed the opposite effect between gene expression and prognosis. It was revealed that the prognosis value of 90.1% DEGs (40/44) was confirmed by using the GEPIA database. In summary, we identified 38 pro-oncogenes and 2 anti-oncogenes in NSCLC.

### Re-analysis of 40 selected DEGs via KEGG pathway enrichment

**Figure 5:**
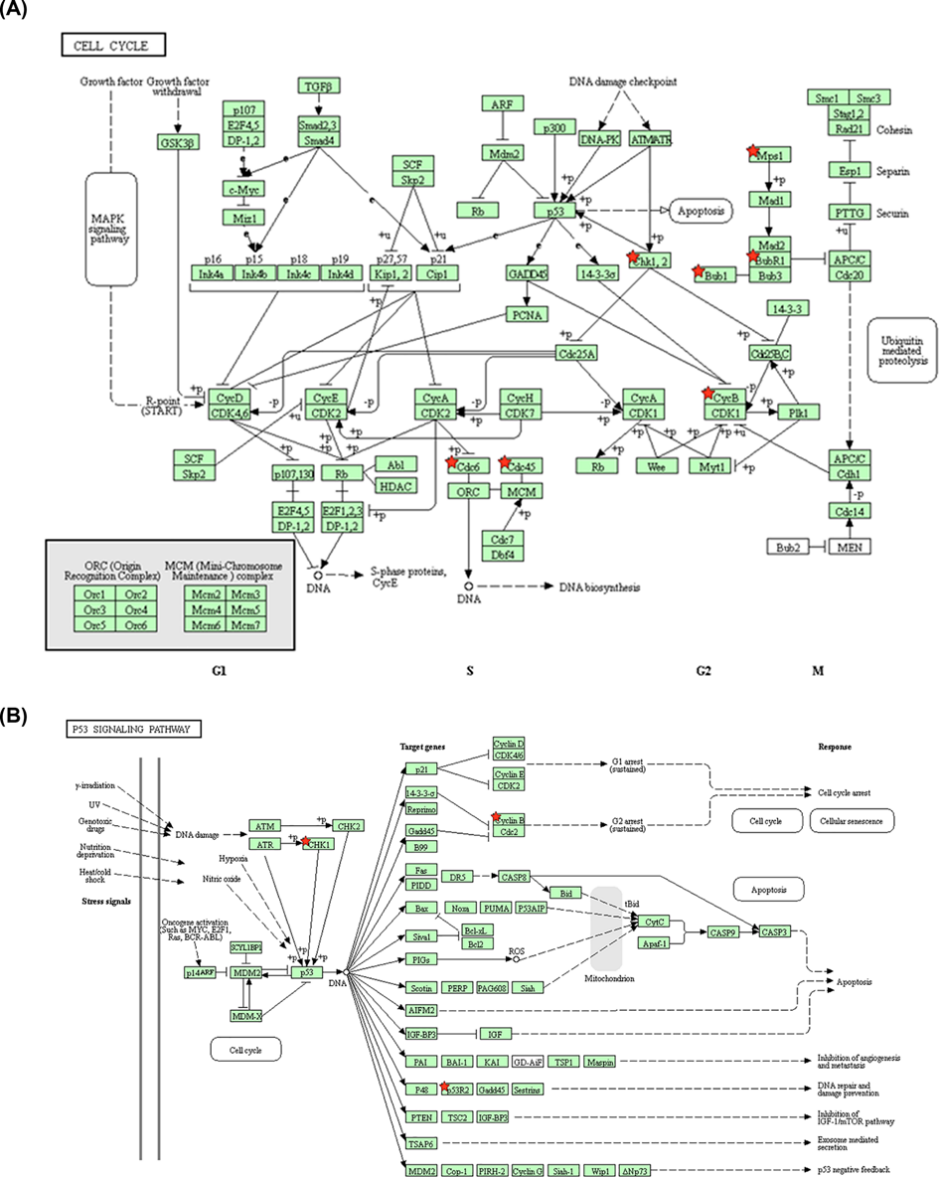
KEGG pathway analysis of 40 core DEGs (**A**) Cell cycle signaling pathway is significantly enriched with BUB1B, BUB1, TTK, CDC45, CDC6, CHECK1, CCNB1, and CCNB2 that were involved in the cell cycle signaling pathway of NSCLC. (**B**) A p53 signaling pathway is significantly enriched with CCNB1, CCNB2, and RRM2 that were revealed in the p53 signaling pathway. The red star show the DEGs show up in cell cycle p53 signaling pathway. The red star indicates the enriched gene.

To further evaluate the possible pathway of these 40 selected core DEGs, KEGG pathway enrichment was re-analyzed via DAVID. Cell cycle, p53 signaling pathway, progesterone-mediated oocyte maturation, and oocyte meiosis signaling pathway were enriched (*P*<0.05; Supplementary Table S8). Finally, we selected cell cycle and p53 signaling pathway as significantly enriched signaling pathway (*P*<0.05, FDR<0.05). Eight DEGs were involved in cell cycle signaling (Figure 5A, BUB1B, BUB1, TTK, CDC45, CDC6, CHECK1, CCNB1, and CCNB2). Moreover, four DEGs were involved in the p53 signaling pathway (Figure 5B, CHEK1, CCNB1, CCNB2, and RRM2). Eventually, the nine overlapped DEGs were TTK, BUB1B, BUB1, CDC45, CDC6, CHECK1, CCNB1, CCNB2, and RRM2, which were all pro-oncogenes. In summary, nine key pro-oncogenes were identified as the biomarkers of prognosis for NSCLC.

### Validation of the protein level of nine key pro-oncogenes

**Figure 6:**
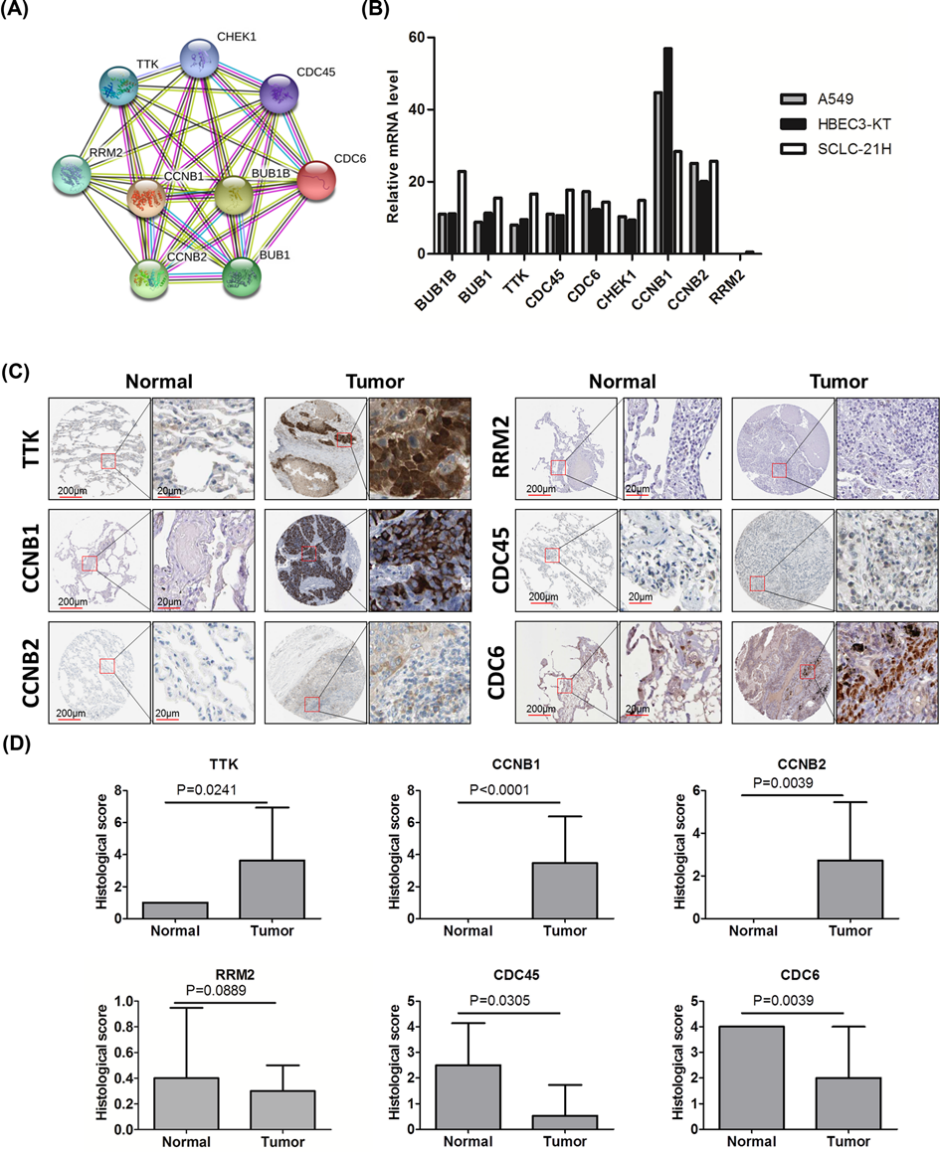
The protein levels of nine key genes in NSCLC (**A**) The PPI network analysis of nine key pro-oncogenes (BUB1B, BUB1, TTK, CDC45, CDC6, CHECK1, CCNB1, CCNB2 and RRM2). (**B**) The mRNA expression of nine key pro-oncogenes in three NSCLC tumor cell lines. (**C**) The represent immunohistochemistry (IHC) images of TTK, CCNB1, CCNB2, RRM2, CDC45, and CDC6. (**D**) The expression of protein in the tissues was scored semi-quantitatively by combining the staining and the intensity (staining index = staining × intensity). Sample size: TTK (11 NSCLC, 3 normal), CCNB1 (34 NSCLC, 5 normal), CCNB2 (34 NSCLC, 5 normal), RRM2 (23 NSCLC, 5 normal), CDC45 (22 NSCLC, 6 normal), and CDC6 (3 NSCLC, 11 normal).

Next, we confirmed the PPI network for these nine key pro-oncogenes. They showed a strong interaction with each other (Figure 6A). As the *in vitro* experiment also very important for mechanism studies, we checked the mRNA expression of nine key pro-oncogenes in A549, HBEC3-KT, and SCLC-21H by using Human Protein Atlas database. These nine genes showed similar expression patterns in these three cell lines, and CCNB1 revealed the highest expression in three cell lines (Figure 6B). Furthermore, we checked the protein levels of these nine genes in Human Protein Atlas. There is no staining of TTK, CCNB1, and CCNB2 in normal human lung tissues; however TTK, CCNB1, and CCNB2 showed strong positive in NSCLC tissues; the majority of positive cells were tumor cells and immune cells (Figure 6C). Furthermore, the semi-quantitative method by combining the staining and the intensity confirmed that TTK, CCNB1, and CCNB2 were significantly increased in NSCLC tissues when compared with that in normal lung tissues (Figure 6D, *P*<0.05). Conversely, the pro-oncogenes RRM2, CDC45, and CDC6 were decreased in NSCLC tissues compared with that in normal tissues (Figure 6C). RRM2 showed no expression in both normal and tumor tissues, while CDC45, and CDC6 were mainly expressed in tumor cells and immune cells (Figure 6C,D). There was no expression data for BUB1B, BUB1, and CHECK1. In summary, we confirmed TTK, CCNB1 and CCNB2 were pro-oncogenes that mainly expressed in tumor cells and immune cells.

### Distribution of nine key pro-oncogenes in single-cell level

**Figure 7:**
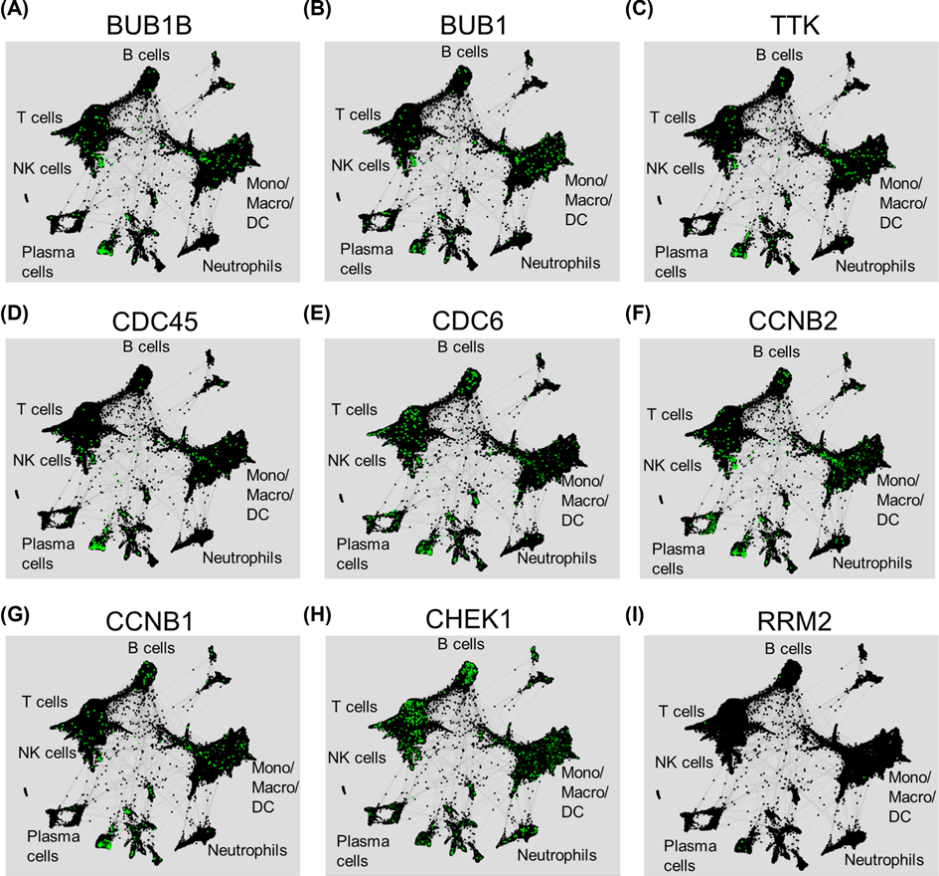
Distribution of nine key pro-oncogenes in single-cell level (**A-I**) Seven lung tumor samples were dissociated for single-cell RNA sequencing and re-clustered into natural killer (NK) cells T cells, B cells, monocyte/macrophage/dendritic cells (DC), neutrophils, plasma cells, and other cells by using specific cell markers. The distribution of nine selected genes in human total tumor cells were retrieved from the website tool. A dark dot means the total number of individual cells; a green dot means the specific cell that expresses the indicated gene.

As the heterogeneity of gene expression in NSCLC showed by IHC, next, we confirmed nine key pro-oncogene in by using a single-cell RNA-sequencing based database. BUB1B, BUB1, and TTK mainly expressed on T cells, NK cells, and monocytes/macrophage/DC cells (Figure 7A–C). The majority of CDC45 expressed on NK cells, and monocytes/macrophage/DC cells (Figure 7D,E). CDC6 and CCNB2 mainly distributed on B cells, T cells, NK cells, monocytes/macrophage/DC cells, and plasma cells (Figure 7F). CCNB1 mainly expressed on B cells, T cells, monocytes/macrophage/DC cells, and NK cells (Figure 7G). CHECK1 expressed on all cell types, and RRM2 showed no expression on all the cells (Figure 7H,I). Similar results were also verified in another single-cell RNA-sequencing based database (Supplementary Figure S2). In summary, we showed the expression of nine key pro-oncogenes in the single-cell level in NSCLC.

### Validation of TTK as a biomarker for prognosis in NSCLC

Of these nine validated pro-oncogenes, we found by bioinformatics analysis, TTK was chosen to verifying its role as a biomarker for prognosis in NSCLC by using a tissue microarray. According to the expression of TTK, 90 cases of NSCLC were divided into the low TTK group and high TTK group with 45 cases in each group. The average levels of TTK in the low TTK group and high TTK group weres 1.98 ± 0.95 and 7.13 ± 1.47, respectively (Table 1). There is no significant difference in age, gender, location, and distant metastasis between the low TTK group and the high TTK group (Table 1, *P*>0.05). Whereas, in the low TTK group, 33 cases (73.3%) were diagnosed at histological stage I-II while 12 cases (26.7%) were diagnosed at stage III-IV. However, in the high TTK group, 20 cases (44.4%) were diagnosed at histological stage I-II while 25 cases (55.6%) were diagnosed at histological stage III-IV, indicating that high TTK expression was related to the advanced histological stage (Table 1, *P*=0.005). Furthermore, in the low TTK group, 28 cases (82.4%) were in TNM stages I-II, and 6 cases (17.60%) were in TNM stages III-IV. Whereas, in the high TTK group, 15(53.6%) were in TNM stages I-II, and 13 (46.4%) were in TNM stages III-IV, indicating that high TTK expression is significantly associated with advanced TNM stage in NSCLC (*P*=0.015, Table 1). Moreover, the rate of patients with positive lymph nodes in the high TTK group was significantly higher than that in the low TTK group (33.3% vs. 57.8%, *P*=0.017, Table 1).

**Table 1:** Relationships between TTK expression and clinicopathological characteristics in 90 NSCLC cases

Characteristics	TTK expression (%)		*P* value
	Low	High	
Total	45	45	
TTK protein levels	1.98 ± 0.95	7.13 ± 1.47	<0.0001
Average years	61.3 ± 8.6	64.1 ± 8.9	
<65	29 (64.4)	25 (55.6)	0.259
≥65	16 (35.6)	20 (44.4)	
Gender			
Male	44 (97.8)	43 (95.6)	0.500
Female	1 (2.2)	2 (4.4)	
Histologic grade			
I∼II	33(73.3)	20 (44.4)	0.005
III∼ IV	12(26.7)	25 (55.6)	
Location (cm)			
Left brain	21(46.7)	26 (57.8)	0.199
Right brain	24 (53.3)	19 (42.2)	
TNM stage			
I∼II	28 (82.4)	15 (53.6)	0.015
III∼ IV	6 (17.6)	13 (46.4)	
Positive lymph node	15 (33.3)	26 (57.8)	0.017
Distant metastasis	0 (0)	1 (2.2)	0.500

**Figure 8:**
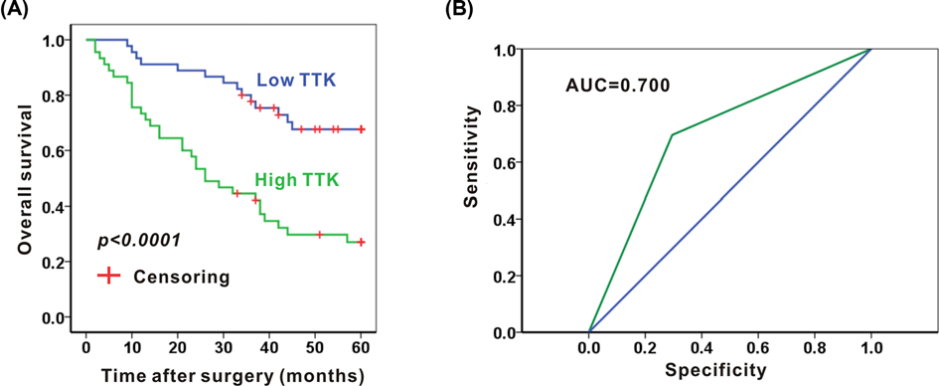
High expression of TTK associates with worse survival in NSCLC (**A**) The 5-year survival was shorter in patients with high TTK expression comparing with those with low TTK expression. (**B**) The ROC curves show the sensitivity and specificity of the TTK signature in predicting the 5-year survival; *N*=45 in each group.

Next, we checked the relationship between expressions of TTK and survival in NSCLC patients. The average five-year overall survival time was 49.7 months for the low TTK group and 31.8 months for the high TTK group. Moreover, the overall 5-year survival rates were 68.9% and 28.9% in the low TTK group and the high TTK group, respectively. Patients with high TTK expression achieved better overall 5-year survival compared with patients with low TTK expression (*P*<0.0001, Figure 8A). Furthermore, we verified the expression of TTK in predicting the prognosis of NSCLC patients by using the ROC curves. The ROC curves indicated the moderate sensitivity and specificity of the TTK signature in predicting the 5-year survival with the AUC 0.700 (Figure 8B). In summary, TTK may serve as a biomarker for prognosis in NSCLC.

## Discussions

Lung cancer is the most common malignancy worldwide. The low 5-years survival (19%) of lung cancer makes it account for 23% of deaths for cancer-related death [18]. Although great effort has been paid for developing a new therapy for lung cancer, the prognosis of lung cancer is still not satisfactory as a lack of early diagnosis. Thus, the new biomarker that either can make the early diagnosis easier or can develop effective therapeutic targets is urgently needed. In the present study, three microarrays of NSCLC that developed in GPL6244 were applied to find new biomarkers. We achieved the following new findings: (1) We identified 284 DEGs may play an important role in the development of NSCLC, with 75 up-regulated DEGs and 209 down-regulated DEGs, these identified DEGs were important for multiple cell processes and cell singling pathways, such as mitotic cell division, angiogenesis, and cell cycle. (2) We selected 40 core DEGs for the development of NSCLC, by showing that nine key genes in the cell cycle and p53 signaling pathway participated in the development of NSCLC. (3) By validated the protein level of nine key genes by semi-quantitative of IHC and checked the distribution in single-cell level, TTK, CCNB1, and CCNB2 were selected as the pro-oncogenes. (4) Experimental validated TTK may serve as a biomarker for prognosis in NSCLC. Although there are some publications about the bioinformatics analysis of NSCLC [19,20], this is the first systematic analysis article that identified key genes for the development of NSCLC and experimental validated TTK as a biomarker of prognosis in NSCLC. Also, this is the first bioinformatics analysis of NSCLC that showing gene expression at a single-cell level. The present study will provide new insights for the development of individualized therapeutic targets for NSCLC.

In the present study, 75 up-regulated DEGs and 209 down-regulated DEGs were identified by bioinformatics analysis of microarray data. However, after using PPI network analysis, Kaplan–Meier plotter and GEPIA, only 2 down-regulated DEGs and 38 up-regulated DEGs were selected as core DEGs. It shows that the up-regulated genes may much more important for the development of NSCLC. These observations are consistent with other bioinformatics analysis in other cancers, such as ovarian cancer [21]. Also, as 90.1% DEGs (40/44) were confirmed by using the Kaplan–Meier plotter and GEPIA, it is predictable that the bioinformatics analyses we used in the present study are effective to find functional genes for NSCLC. Other DEGs may also have some effect on the development and prognosis of NSCLC, which need further investigating.

In cancers, defects in cell cycle and checkpoint attribute gene mutations, chromosome damages which can eventually contribute to altered cell proliferation and tumorigenesis [22]. In the GO analysis, most of the biology processes are related the cell proliferation and checkpoint. It is presumable that the DEGs may mainly involve in cell proliferation and checkpoint abnormal in NSCLC. This hypothesis is also confirmed by the KEGG pathway enrichment, which revealed that cell cycle and p53 signaling pathways are the significant enriched signaling pathway. Cell cycle signaling pathway is important for the development of NSCLC, thus several cell cycle inhibitors have been applied in the treatment of NSCLC [23–25]. CDK4/6 inhibitor palbociclib induces cell cycle arrest at the G1/S phase transition and consequently reduces tumor growth [25]. In the present study, we found that TTK, BUB1B, BUB1, CDC45, CDC6, CHECK1, CCNB1, and CCNB2 were involved in the cell cycle signaling pathway of NSCLC. P53 transcriptional activation potential is critical for suppressing cancer. The p53 signaling pathway also plays a pivotal role in the development of NSCLC [26,27]. The p53 target gene SIVA can regulate both metabolism and tumorigenesis of NSCLC. Consistently, the p53 signaling pathway was also significantly enriched in the present study. We found that CHEK1, CCNB1, CCNB2, and RRM2 were revealed in the p53 signaling pathway. Thus BUB1B, BUB1, TTK, CDC45, CDC6, CHECK1, CCNB1, CCNB2, and RRM2 may serve as the new prognostic biomarker and may also identify as a potential target for NSCLC cancer therapy.

Most genes exert their function by translating into specific proteins. We found that the protein of TTK, CCNB1, and CCNB2 much stronger expressed in tumor cells and immune cells in NSCLC when compared with that in normal lung tissues. However, the pro-oncogenes RRM2, CDC45, and CDC6 were decreased in NSCLC tissues compared with that in normal tissues, which showed opposite trends with mRNA levels. The low expression of mRNA and small sample size for IHC may attribute to the discrepancy between the expression of mRNA and protein. Dual specificity protein kinase TTK plays key roles in meiosis, cytokinesis, spindle pole assembly, and spindle checkpoint [28]. However, less attention is paid for TTK in NSCLC. There is only one publication demonstrated that TTK is the substrate for X-linked deubiquitinas (USP9X) [28]. The protein expression levels of TTK were increased in NSCLC tumor tissues and inhibition of TTK reduces A549 cell proliferation, migration and tumorigenesis [28]. Thus, we next validated effect of TTK in prognosis in NSCLC by using a tissue microarray that contains 90 cases of NSCLC. As expected, high TTK expression associated with higher histological stage, advanced TNM stage, high frequency of positive lymph nodes and worse 5-year overall survival. In accordance with our experimental results, our bioinformatics analysis also confirmed increased mRNA and protein levels of TTK in NSCLC and high expression of TTK correlates with poor prognosis. Moreover, TTK mainly distributes on T cells, NK cells, and monocytes/macrophage/DC cells. Thus, TTK may play a critical role in NSCLC, and could be considered as a potential therapeutic target and biomarker for the prognosis of NSCLC.

In conclusion, we identified 284 DEGs that may play a critical role in the development of NSCLC. Forty core DEGs correlates with prognosis and nine key genes were enriched in cell cycle and p53 signaling pathway. Finally, we validate TTK as the pro-oncogenes that may play a pivotal role in NSCLC, and could be considered as a potential therapeutic target and biomarker for the prognosis of NSCLC. Collectively, the present study provides new insights for the development of individualized therapeutic targets for NSCLC.

## Supplementary Material

Supplementary Figures S1-S3 and Tables S1-S8

## Data Availability

All the data are present in the manuscript. And the datasets is available at NCBI GEO database with the accession number GSE31552, GSE43458 and GSE44077. All the data are available from the corresponding author Yuecan Zeng (Zeng.yuecan@hotmail.com) under reasonable request.

## Abbreviations

DEG: differentially expressed gene
IHC: immunohistochemistry
NSCLC: non-small cell lung cancer
PPI: protein–protein interaction
