# Supplementary material for: Bioinformatics analysis and experimental validation of TTK as a biomarker for prognosis in non-small cell lung cancer
Source: Biosci Rep. 2020 Oct 6;40(10):BSR20202711. doi: 10.1042/BSR20202711 (PMC7538683; doi:10.1042/BSR20202711)
Supplement: Supplementary Figures S1-S3 and Tables S1-S8 [file BSR-2020-2711_supp.pdf]

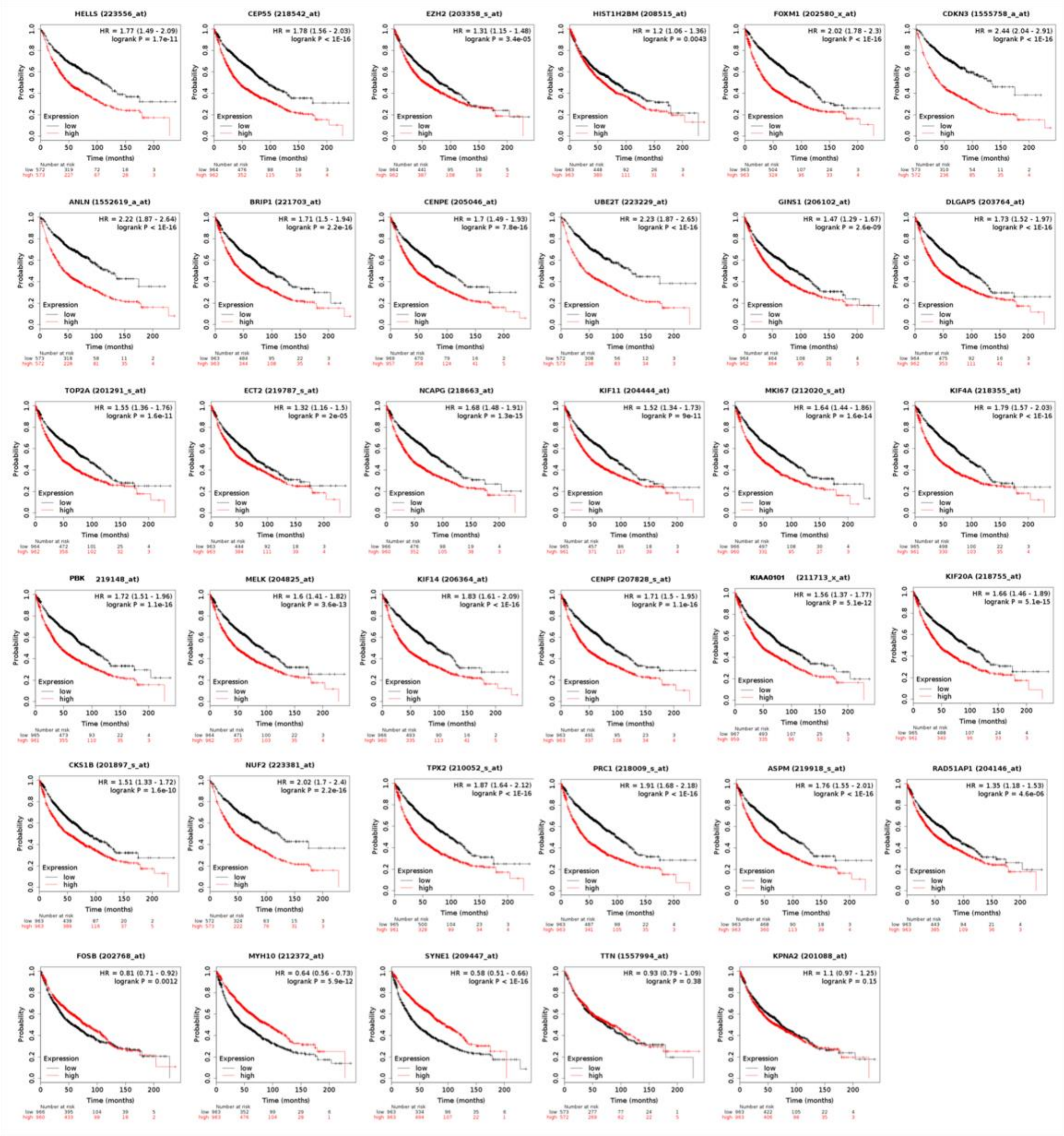
HELLS (223556_at)
HR = 1.77 (1.49 - 2.09) logrank P = 1.7e-11
CEP55 (218542_at)
HR = 1.78 (1.56 - 2.03) logrank P < 1E-16
EZH2 (203358_s_at)
HR = 1.31 (1.15 - 1.48) logrank P = 3.4e-05
HIST1H2BM (208515_at)
HR = 1.2 (1.06 - 1.36) logrank P = 0.0043
FOXM1 (202580_x_at)
HR = 2.02 (1.78 - 2.3) logrank P < 1E-16
CDKN3 (1555758_a_at)
HR = 2.44 (2.04 - 2.91) logrank P < 1E-16
ANLN (1552619_a_at)
HR = 2.22 (1.87 - 2.64) logrank P < 1E-16
BRIP1 (221703_at)
HR = 1.71 (1.5 - 1.94) logrank P = 2.2e-16
CENPE (205046_at)
HR = 1.7 (1.49 - 1.93) logrank P = 7.8e-16
UBE2T (223229_at)
HR = 2.23 (1.87 - 2.65) logrank P < 1E-16
GINS1 (206102_at)
HR = 1.47 (1.29 - 1.67) logrank P = 2.6e-09
DLGAP5 (203764_at)
HR = 1.73 (1.52 - 1.97) logrank P < 1E-16
TOP2A (201291_s_at)
HR = 1.55 (1.36 - 1.76) logrank P = 1.6e-11
ECT2 (219787_s_at)
HR = 1.32 (1.16 - 1.5) logrank P = 2e-05
NCAPG (218663_at)
HR = 1.68 (1.48 - 1.91) logrank P = 1.3e-15
KIF11 (204444_at)
HR = 1.52 (1.34 - 1.73) logrank P = 9e-11
MKI67 (212020_s_at)
HR = 1.64 (1.44 - 1.86) logrank P = 1.6e-14
KIF4A (218355_at)
HR = 1.79 (1.57 - 2.03) logrank P < 1E-16
PBK 219148_at)
HR = 1.72 (1.51 - 1.96) logrank P = 1.1e-16
MELK (204825_at)
HR = 1.6 (1.41 - 1.82) logrank P = 3.6e-13
KIF14 (206364_at)
HR = 1.83 (1.61 - 2.09) logrank P < 1E-16
CENPF (207828_s_at)
HR = 1.71 (1.5 - 1.95) logrank P = 1.1e-16
KIAA0101 (211713_x_at)
HR = 1.56 (1.37 - 1.77) logrank P = 5.1e-12
KIF20A (218755_at)
HR = 1.66 (1.46 - 1.89) logrank P = 5.1e-15
CKS1B (201897_s_at)
HR = 1.51 (1.33 - 1.72) logrank P = 1.6e-10
NUF2 (223381_at)
HR = 2.02 (1.7 - 2.4) logrank P = 2.2e-16
TPX2 (210052_s_at)
HR = 1.87 (1.64 - 2.12) logrank P < 1E-16
PRC1 (218009_s_at)
HR = 1.91 (1.68 - 2.18) logrank P < 1E-16
ASPM (219918_s_at)
HR = 1.76 (1.55 - 2.01) logrank P < 1E-16
RAD51AP1 (204146_at)
HR = 1.35 (1.18 - 1.53) logrank P = 4.6e-06
FOSB (202768_at)
HR = 0.81 (0.71 - 0.92) logrank P = 0.0012
MYH10 (212372_at)
HR = 0.64 (0.56 - 0.73) logrank P = 5.9e-12
SYNE1 (209447_at)
HR = 0.58 (0.51 - 0.66) logrank P < 1E-16
TTN (1557994_at)
HR = 0.93 (0.79 - 1.09) logrank P = 0.38
KPNA2 (201088_at)
HR = 1.1 (0.97 - 1.25) logrank P = 0.15
Probability
Time (months)
Expression
low
high
Number at risk

Supplementary Figure S1. Overall survival of 35 core DEGs in NSCLC

Kaplan Meier plotter is applied to generating the survival curve for all 35 core DEGs. Log-rank P value and hazard ratio (HR) with 95% confidence intervals are computed and showed on the plot. Log-rank P<0.05 is considered to be statistical significant.

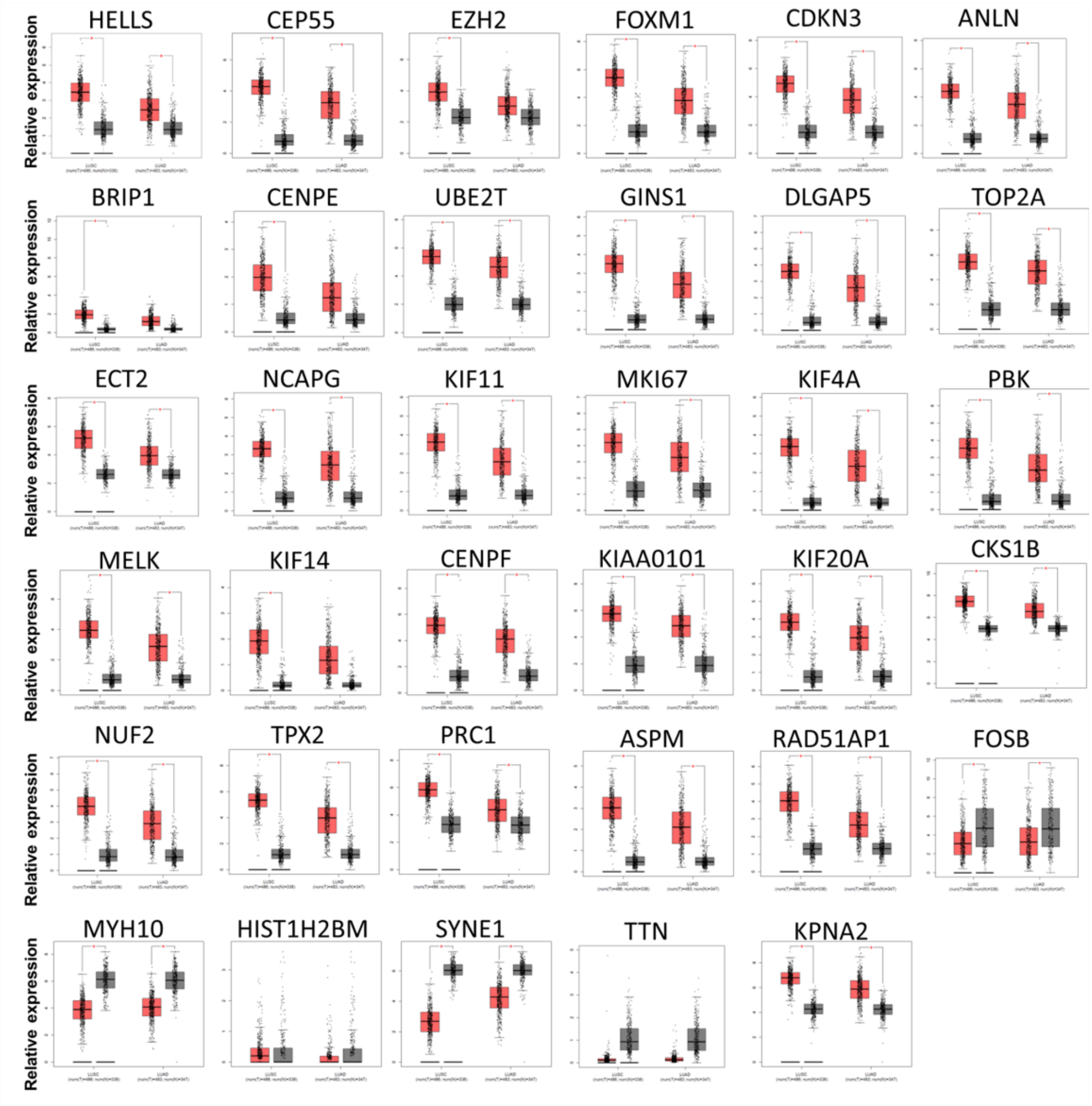
HELLS
CEP55
EZH2
FOXM1
CDKN3
ANLN
BRIP1
CENPE
UBE2T
GINS1
DLGAP5
TOP2A
ECT2
NCAPG
KIF11
MKI67
KIF4A
PBK
MELK
KIF14
CENPF
KIAA0101
KIF20A
CKS1B
NUF2
TPX2
PRC1
ASPM
RAD51AP1
FOSB
MYH10
HIST1H2BM
SYNE1
TTN
KPNA2
Relative expression

Supplementary Figure S2. The mRNA levels of 35 core DEGs in LUAD, LUSC and the corresponding normal lung tissues

30 DEGs are up-regulated, 4 DEGs are downregulated and HIST1H2BM shows no significant differences in LUAD and LUSC compared to corresponding normal lung tissues. The red color histogram on left reveals LUSC, the red color histogram on right shows LUAD, and the gray color histogram means the corresponding normal lung tissues. *P<0.05.

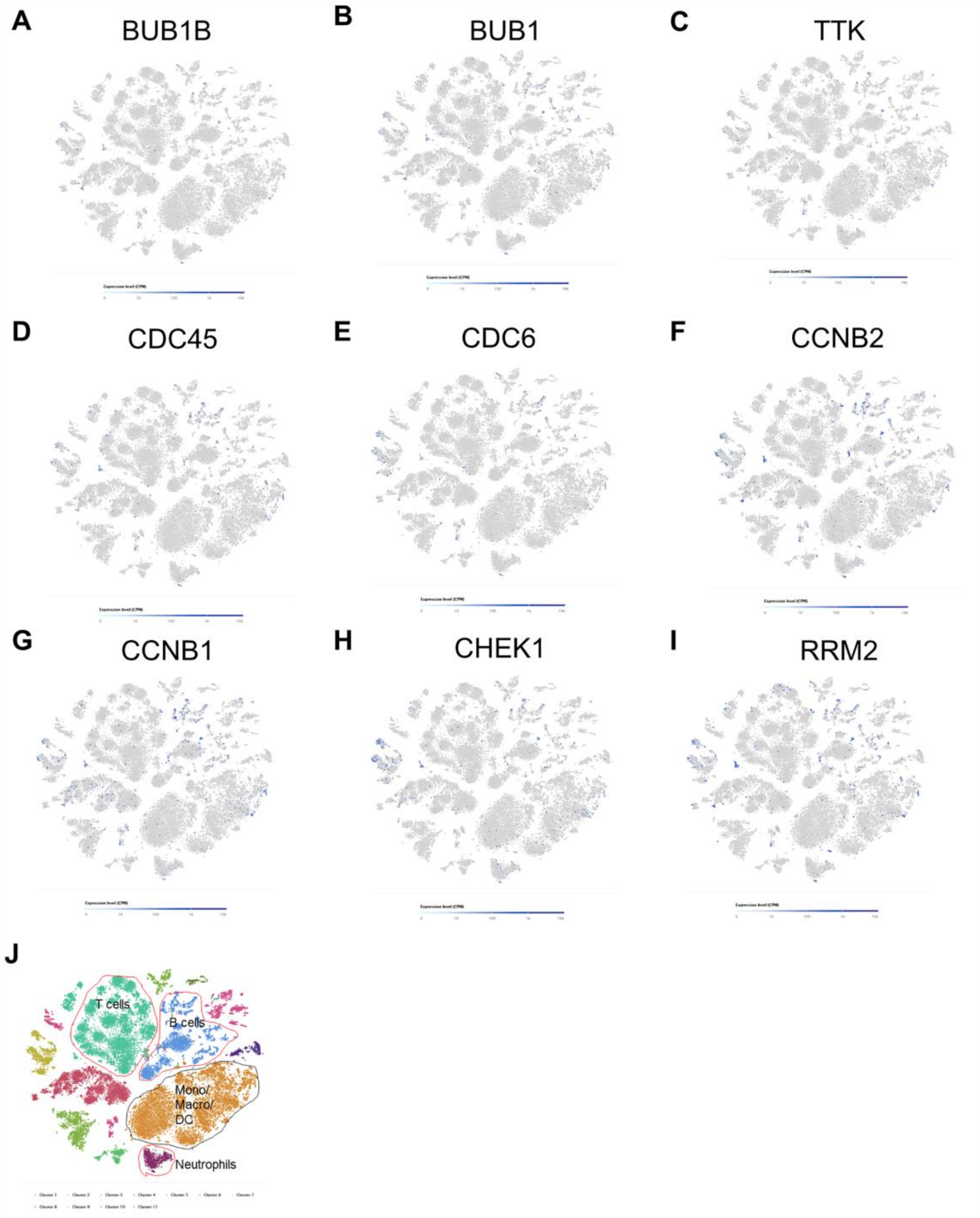
A
BUB1B
B
BUB1
C
TTK
D
CDC45
E
CDC6
F
CCNB2
G
CCNB1
H
CHEK1
I
RRM2
J
T cells
B cells
Mono/
Macro/
DC
Neutrophils

Supplementary Figure S3. Distribution of 9 key pro-oncogenes in single-cell level

3 lung tumor samples were dissociated for single-cell RNA sequencing and re-clustered into natural killer (NK) cells, T cells, B cells, monocyte/macrophage/dendritic cells (DC), neutrophils by using specific cell markers. The distribution of 9 selected genes in human total tumor cells were retrieved from the website tool. The grey dot means the total number of individual cells; the blue dot means the specific cell that expresses the indicated gene.

Supplementary Table 1 Characterize of 3 GEO datasets

Dataset	Tissue type	Platform	Normal	Tumor
**GSE31552**	Adenocarcinomas/ squamous cell carcinoma	GPL6244	57	57
**GSE43458**	Adenocarcinomas	GPL6244	30	80
**GSE44077**	Adenocarcinomas/ squamous cell carcinoma	GPL6244	18	18

Supplementary Table 2 Commonly DEGs among GSE31552, GSE43458 and GSE44077

DEGs	Official gene name
**Up-regulated gene (75)**	RRM2, PBK, GOLM1, FOXM1, GCNT3, GJB2, CDC45, KIF20A, CST1, KIF14, BRIP1, CDKN3, EZH2, CENPE, GALNT7, CHRNA5, KPNA2, CCNB2, GREM1, NME1-NME2///NME1, CDH3, HELLS, RAD51AP1, CKS1B, GSDMC, TTK, TMPRSS11E, GPR87, BUB1, CHEK1, ECT2, NCAPG, KIF4A, PSAT1, KIF11, PRC1, ABCA12, CEP55, HIST1H2BM, MKI67, KIAA0101, CCNB1, SLC7A11, CYP24A1, MELK, CRABP2, OCIAD2, HMGB3, CEACAM5, BUB1B, CDC6, ANKRD22, NUF2, GINS1, UBE2T, FERMT1, SLC2A1, AKR1B10, DSP, ASPM, TPX2, DLGAP5, MMP13, CKMT1A///CKMT1B, , AFAP1-AS1, NQO1, ANLN, MMP1, CENPF, SPINK1, TOP2A, TMPRSS4, MMP12, GPX2, SPP1
**Down-regulated gene (209)**	SFTPC, FABP4, WIF1, ANKRD1, TMEM100, AGER, AQP4, SLC6A4, HBB, FMO2, ABI3BP, FCN3, FIGF, ADH1B, PTPRB, SCN7A, ACADL, TEK, LRRK2, FHL1, RTKN2, CD36, LDB2, EMCN, CALCRL, MRC1, C7, EDNRB, LYVE1, NOSTRIN, PGC, HBA2///HBA1, GKN2, PDK4, EPAS1, TCF21, PECAM1, MFAP4, SFTPA2///SFTPA1, CLDN18, CAV1, NCKAP5, CPB2, IL1RL1, LIFR, HHIP, A2M, HIGD1B, ADGRL2, IGSF10, FOSB, OLR1, PEBP4, CYP4B1, RHOJ, SLIT2, SLCO2A1, ITGA8, LAMP3, ANOS1, MAMDC2, CLIC5, ST8SIA6, AOC3, ABCA8, SLC39A8, NDNF, SVEP1, MYZAP///GCOM1, SPARCL1, SDPR, LOC102725104, COL6A6, MYCT1, PLEKHH2, TNNC1, PKHD1L1, MSR1, ABCA6, GPM6A, LOC102725104, SYNE1, TTN, VEPH1, EMP2, VSIG4, CDH5, GPX3, RGCC, LPL, FHL5, PREX2, PLA2G1B, FBLN5, GNG11, MME, HSD17B6, TIMP3, ANGPT1, CCDC141, PTPRM, ARHGAP29, GIMAP4, LINC00312, RAMP2, SEMA6A, NEXN, CGNL1, KLF4, CHRDL1, PALMD, ADGRF5, PDE5A, DOCK4, GIMAP1-GIMAP5///GIMAP5, CACNA2D2, ARHGEF6, LIMCH1, DPYSL2, WWC2, EDN1, NECAB1, IL7R, FAM189A2, MYH10, CAB39L, SNX25, JAM2, GRIA1, AKAP12, OGN, EPB41L2, HBEGF, GLIPR2, FILIP1, MYADM, SFTPD, BMP5, CFLAR, ADAMTS1, NPNT, ABCC9, ZNF385B, CNTNAP3B, , S1PR1, GIMAP8, KIAA1324L, FAM184A, PRG4, GRK5, SULT1C4, CXCL2, ANGPTL1, SASH1, CCDC68, F8, MMRN1, ITM2A, LRRC36, PDLIM3, DACH1, DPT, PPARG, ARHGEF26, IL18R1, RPL23AP32, FLI1, GLDN, ASPA, TMOD1, ADAMTSL3, MIR30C2, ACVRL1, PAPSS2, RANBP3L, OTUD1, RBMS3, LOC102725104, SPTBN1, ZFP36, GBP4, AFF3, KHDRBS2, LHFP, TREM1, ACSS3, TACC1, SOGA3///KIAA0408, DNAJB4, HECW2, LRRFIP1, LRP2, MARCO, TRHDE, MEIS1, MSRB3, GIMAP7, FZD4, STXBP6, SCEL, ERG, ANKRD20A8P, ADARB1, LOC102725104///ANKRD20A8P, CLEC3B, F11, ROBO2, FERMT2, PDE1C

Supplementary Table 3 Gene ontology analysis of up-regulated commonly DEGs among GSE31552, GSE43458 and GSE44077

Category	Term	Count	%DEGs	P-Value	FDR
**GOTERM_BP_DIRECT**	mitotic nuclear division	13	17.3	3.50E-10	1.80E-07
**GOTERM_BP_DIRECT**	cell division	14	18.7	1.50E-09	3.90E-07
**GOTERM_BP_DIRECT**	mitotic spindle assembly checkpoint	4	5.3	7.20E-05	1.20E-02
**GOTERM_BP_DIRECT**	chromosome segregation	5	6.7	1.70E-04	2.20E-02
**GOTERM_BP_DIRECT**	mitotic cytokinesis	4	5.3	2.20E-04	2.30E-02
**GOTERM_BP_DIRECT**	G2/M transition of mitotic cell cycle	6	8.0	2.50E-04	2.10E-02
**GOTERM_BP_DIRECT**	mitotic cell cycle checkpoint	4	5.3	3.00E-04	2.20E-02
**GOTERM_BP_DIRECT**	microtubule-based movement	5	6.7	3.40E-04	2.20E-02
**GOTERM_BP_DIRECT**	DNA replication	6	8.0	4.40E-04	2.50E-02
**GOTERM_BP_DIRECT**	mitotic metaphase plate congression	4	5.3	4.70E-04	2.40E-02
**GOTERM_BP_DIRECT**	cell proliferation	8	10.7	7.50E-04	3.40E-02
**GOTERM_CC_DIRECT**	nucleoplasm	26	34.7	2.00E-05	1.30E-03
**GOTERM_CC_DIRECT**	cytoplasm	37	49.3	4.90E-05	2.10E-03
**GOTERM_CC_DIRECT**	kinesin complex	5	6.7	5.10E-05	1.60E-03
**GOTERM_CC_DIRECT**	spindle pole	6	8.0	6.20E-05	1.60E-03
**GOTERM_CC_DIRECT**	chromosome, centromeric region	5	6.7	6.80E-05	1.40E-03
**GOTERM_CC_DIRECT**	spindle	6	8.0	1.00E-04	1.80E-03
**GOTERM_CC_DIRECT**	cytosol	27	36.0	1.40E-04	2.20E-03
**GOTERM_CC_DIRECT**	kinetochore	5	6.7	2.70E-04	3.70E-03
**GOTERM_CC_DIRECT**	Condensed chromosome outer kinetochore	3	4.0	4.00E-04	5.00E-03
**GOTERM_CC_DIRECT**	membrane	20	26.7	4.70E-04	5.40E-03
**GOTERM_CC_DIRECT**	nucleus	35	46.7	6.80E-04	7.10E-03
**GOTERM_CC_DIRECT**	spindle midzone	3	4.0	2.40E-03	2.30E-02
**GOTERM_CC_DIRECT**	condensed chromosome kinetochore	4	5.3	4.60E-03	4.00E-02
**GOTERM_MF_DIRECT**	ATP binding	19	25.3	1.40E-05	2.30E-03
**GOTERM_MF_DIRECT**	microtubule motor activity	5	6.7	2.90E-04	2.30E-02
**GOTERM_MF_DIRECT**	protein kinase binding	8	10.7	7.10E-04	3.80E-02
**GOTERM_MF_DIRECT**	protein binding	49	65.3	8.30E-04	3.40E-02
**GOTERM_MF_DIRECT**	microtubule binding	6	8.0	1.40E-03	4.60E-02

Supplementary Table 4 Gene ontology analysis of down-regulated commonly DEGs among GSE31552, GSE43458 and GSE44077

Category	Term	Count	%DEGs	P-Value	FDR
**GOTERM_BP_DIRECT**	angiogenesis	12	5.7	2.90E-07	2.60E-04
**GOTERM_BP_DIRECT**	regulation of blood pressure	6	2.9	3.40E-05	1.50E-02
**GOTERM_BP_DIRECT**	cell adhesion	10	4.8	1.40E-04	4.10E-02
**GOTERM_CC_DIRECT**	membrane raft	9	4.3	1.90E-05	3.10E-03
**GOTERM_CC_DIRECT**	cell surface	14	6.7	4.20E-05	3.50E-03
**GOTERM_CC_DIRECT**	extracellular space	25	12.0	4.30E-05	2.40E-03
**GOTERM_CC_DIRECT**	extracellular exosome	42	20.1	6.40E-05	2.70E-03
**GOTERM_CC_DIRECT**	proteinaceous extracellular matrix	10	4.8	1.00E-04	3.40E-03
**GOTERM_CC_DIRECT**	sarcomere	4	1.9	4.50E-04	1.20E-02
**GOTERM_MF_DIRECT**	heparin binding	9	4.3	1.60E-05	4.10E-03

Supplementary Table 5 KEGG pathway analysis of commonly DEGs among GSE31552, GSE43458 and GSE44077

DEGs	Pathway ID	Count	%DEGs	P-Value	FDR	Genes
Up-regulated	Cell cycle	8	10.7	3.30E-07	1.70E-05	BUB1B, BUB1, TTK, CDC45, CDC6, CHEK1, CCNB1, CCNB2
	p53 signaling pathway	4	5.3	2.40E-03	6.20E-02	CHEK1, CCNB1, CCNB2, RRM2
	Progesterone-mediated oocyte maturation	3	4.0	4.40E-02	5.50E-01	BUB1, CCNB1, CCNB2
	Oocyte meiosis	3	4.0	7.00E-02	6.10E-01	BUB1, CCNB1, CCNB2
Down-regulated	PPAR signaling pathway	6	2.9	8.30E-04	9.90E-02	CD36, ACADL, FABP4, LPL, OLR1, PPARG
	Complement and coagulation cascades	5	2.4	7.70E-03	3.80E-01	A2M, CPB2, F8, F11, C7
	Cell adhesion molecules (CAMs)	6	2.9	2.30E-02	6.30E-01	CDH5,CLDN18, ITGA8, JAM2, PECAM1, PTPRM
	Phagosome	6	2.9	2.60E-02	5.60E-01	CD36, MARCO, MSR1, MRC1, OLR1, SFTPD

Supplementary Table 6 The prognostic effect of 44 central node genes

Category	Genes
**Gene high expression with significantly worse survival (39, P<0.05)**	HELLS, RRM2, CEP55, EZH2, HIST1H2BM, FOXM1, CDC45, CHEK1, CDKN3, ANLN, BRIP1, CENPE, UBE2T, GINS1, DLGAP5, TOP2A, ECT2, NCAPG, CCNB2, KIF11, MKI67, KIF4A, PBK, MELK, KIF14, BUB1B, CENPF, KIAA0101, CCNB1, KIF20A, BUB1, CKS1B, NUF2, TTK, CDC6, TPX2, PRC1, ASPM, RAD51AP1
**Gene low expression with significantly worse survival (3, P<0.05)**	FOSB, MYH10, SYNE1,
**Gene expression with no effect on survival (2, P>0.05)**	KPNA2, TTN

Supplementary Table 7 Validation of 42 selected gene expression in NSCLC and normal tissue

Category	Genes
**Gene high expression with significantly worse survival and high expression in tumor tissue (38)**	HELLS, RRM2, CEP55, EZH2, FOXM1, CDC45, CHEK1, CDKN3, ANLN, BRIP1, CENPE, UBE2T, GINS1, DLGAP5, TOP2A, ECT2, NCAPG, CCNB2, KIF11, MKI67, KIF4A, PBK, MELK, KIF14, BUB1B, CENPF, KIAA0101, CCNB1, KIF20A, BUB1, CKS1B, NUF2, TTK, CDC6, TPX2, PRC1, ASPM, RAD51AP1
**Gene low expression with significantly worse survival high expression in normal tissue (2)**	FOSB, MYH10,
**Exclude genes with opposite effect between prognosis and expression (1)**	SYNE1
**Exclude genes with no significantly change between NSCLC and normal tissue (1, P>0.05)**	HIST1H2BM

Supplementary Table 8 KEGG pathway analysis of 40 validated genes

Pathway ID	Count	%DEGs	P-Value	FDR	Genes
**Cell cycle**	8	20.0	1.60E-09	3.20E-08	BUB1B, BUB1, TTK, CDC45, CDC6, CHEK1, CCNB1, CCNB2
**p53 signaling pathway**	4	10.0	3.00E-04	3.00E-03	CHEK1, CCNB1, CCNB2, RRM2
**Progesterone-mediated oocyte maturation**	3	7.5	1.30E-02	8.40E-02	BUB1, CCNB1, CCNB2
**Oocyte meiosis**	3	7.5	2.10E-02	9.90E-02	BUB1, CCNB1, CCNB2
